# Supplementary material for: Annual time-series analysis of aqueous eDNA reveals ecologically relevant dynamics of lake ecosystem biodiversity
Source: Nat Commun. 2017 Jan 18;8:14087. doi: 10.1038/ncomms14087 (PMC5253663; doi:10.1038/ncomms14087)
Supplement: Supplementary Information — Supplementary Figures, Supplementary Tables, Supplementary Methods and Supplementary References [file ncomms14087-s1.pdf]

# Supplementary Methods

**Emergence patterns of Chironomidae & the Chironomid Pupal Exuviae Technique (CPET).** Chironomids exhibit specialised responses to ecological stressors and are acknowledged as one of the most important macroinvertebrate groups for monitoring lake ecosystem health [1]. However, benthic larvae collected with traditional kick-net sampling are notoriously difficult to identify, even by specialists. To overcome these problems lentic Chironomidae biodiversity is assessed via the identification of shed exuviae (skins) of emerging adults that float and accumulate on the leeward edge of lentic ecosystems [1,2]. Exuvial samples therefore offer a unique advantage to simultaneously compare the diversity of recent lentic invertebrate communities and eDNA, and to explore how eDNA is related to ecosystem wide biodiversity. Additionally, using the CPET technique, compared to traditional kick-net sampling, allows for integrated collection of specimens from a wide range of habitats rather than only the profundal zone. The collection and sorting process is fast and the identification of the exuviae is easier than identification of larvae, while the sample collected is also fresh, as the exuviae remain floating for only about 48h [1].

The emergence patterns of Chironomidae are known to differ in different latitudinal zones, due to variations in temperature and photoperiod [3]. In the tropics, the emergence cycles are accelerated, following the lunar cycles, with species emerging all year round. On the contrary, closer to the Arctic, emergence of adults occurs over a limited window over the summer period. Emergence is limited also by surface freezing of the water bodies. For the temperate zones, emergence is higher over the summer but not limited to that time. Species are known to emerge across all seasons, but with less intensity in winter months.

Hence an episodic pattern occurs, with lower emergence over winter, which increases gradually over time.

**Testing of capture and extraction protocols for eDNA**. Rigorous testing of eDNA capture and extraction protocols was performed prior to commencing the experiment. For testing of filtration methods, two types of filtration membranes at different pore sizes were used: glass fibre at 0.7µm and cellulose nitrate at 0.45µm and 0.2µm. Two volumes of water samples were used at 1L and 2L. Ethanol precipitation and centrifugation, using 15ml water samples was also tested, as well as direct centrifugation of 50ml water samples (no precipitation or filtration). For the latter two, varying centrifugation speeds and centrifugation times were also tested. The extraction protocols included the DNeasy Blood & Tissue kit (QIAGEN), Power Water DNA Isolation kit (MoBio) and Phenol Chloroform extraction protocol (PCI) as per [4] with an added Proteinase K step.

From all the above, the collection of eDNA using 0.45µm cellulose filter membranes (2lt water) coupled with a PCI extraction protocol was considered optimal, due to the following: (1) Higher concentrations of collected DNA as per spectrophotometric quantification (NanoDrop) and quality of DNA from agarose gel visualization. (2) Possibility for collection of larger water sample (2L). (3) Ease of storage of collected samples (filter membrane) until DNA extraction (storage at -80$^{o}$C). (4) Optimal pore size for collection of smaller DNA molecules (compared to glass fibre 0.7µm) and filtration time efficiency (compared to cellulose 0.2µm). (5) Good performance in PCR amplification of long COI amplicons.

## Supplementary Figures

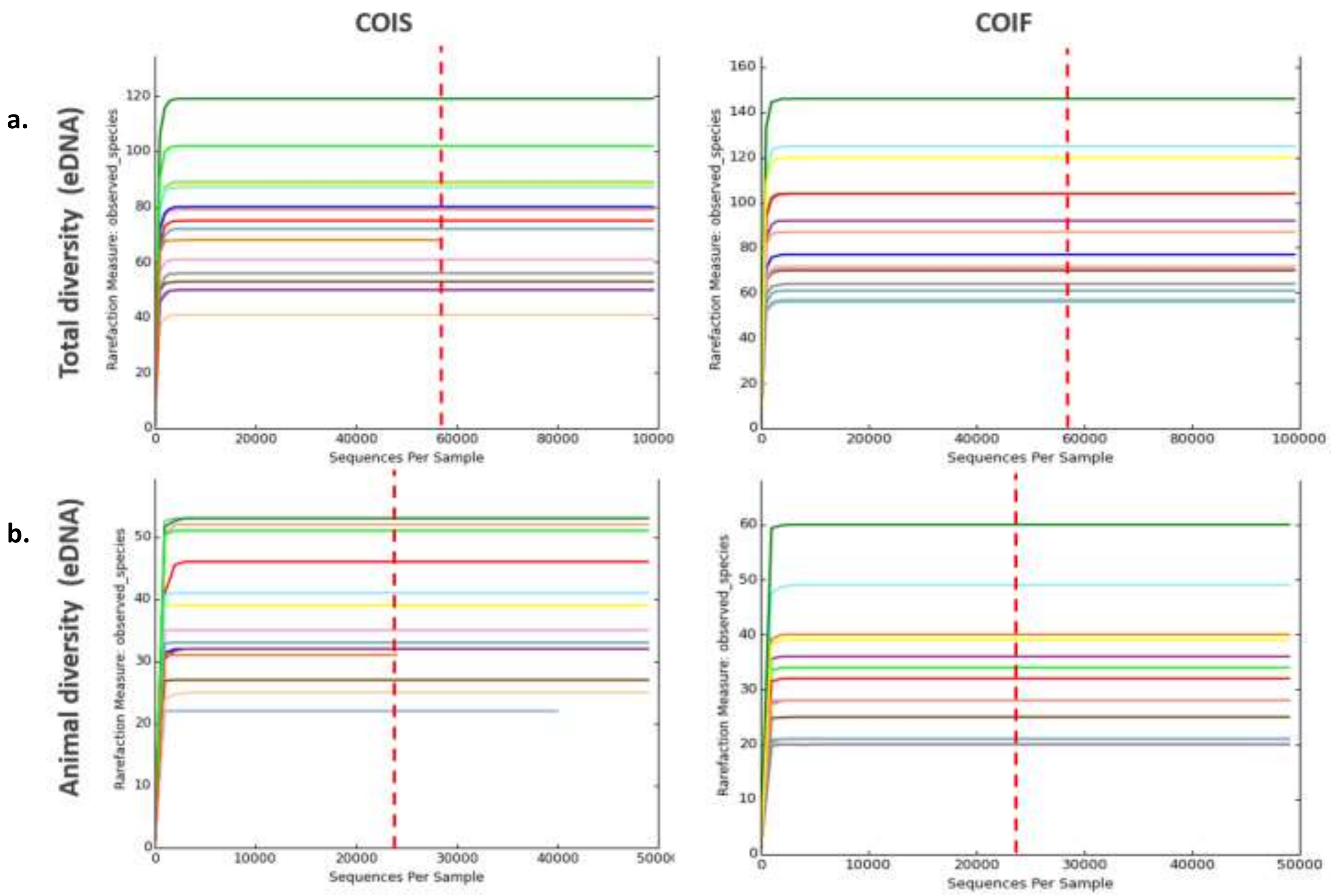


**Supplementary Figure 1 | Rarefaction plots**. The figure shows (a) total taxa and (b) animal taxa only, based on water extracted eDNA samples only for both amplicons (COIS and COIF). Dashed red lines indicate the rarefaction depth used for analysis (a. total taxa 57,869 reads, b. animal taxa 24,914 reads), x-axis: reads per sample, y-axis: OTU richness (N = 64).

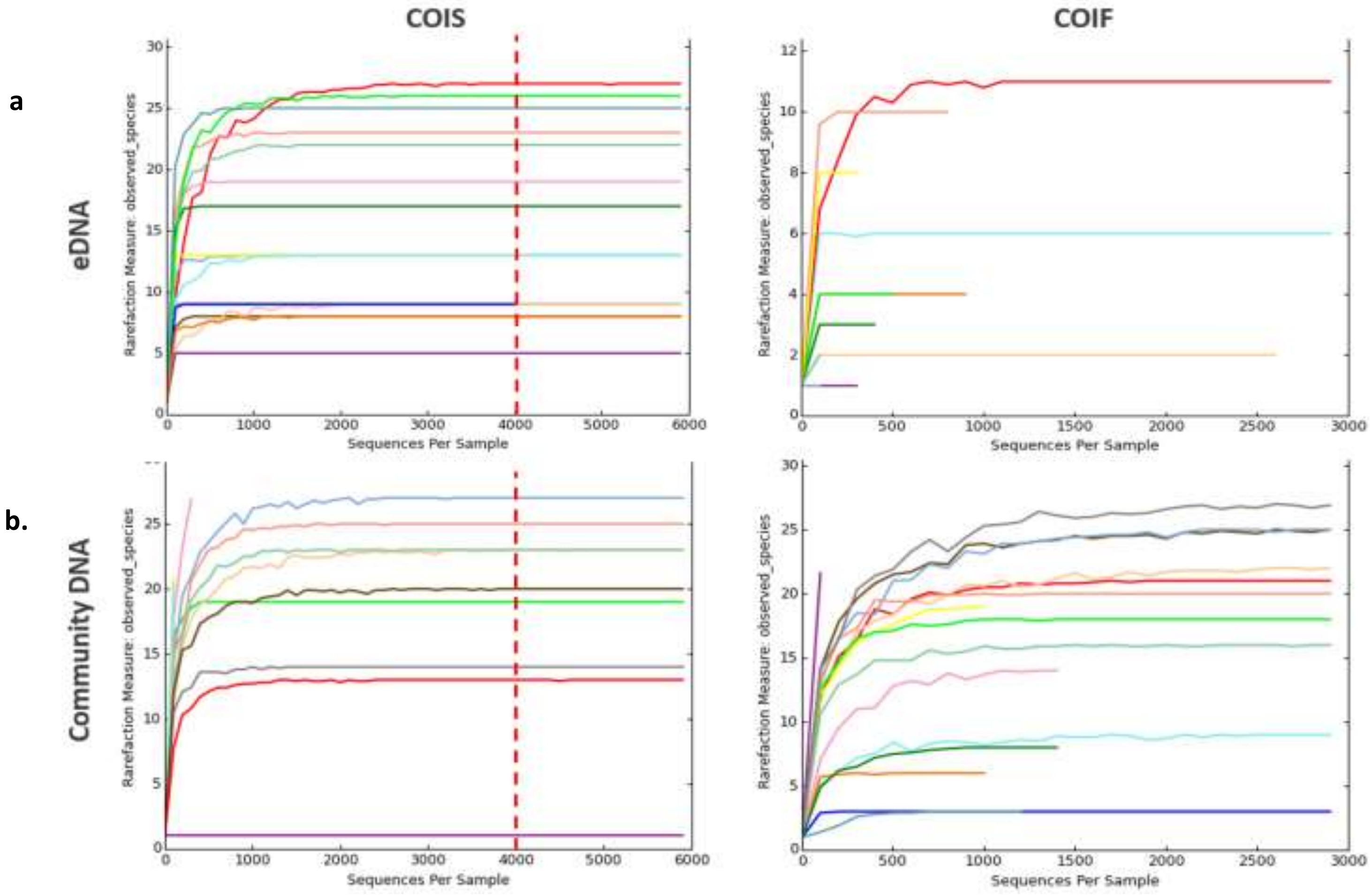


**Supplementary Figure 2 | Rarefaction plots.** The figure shows Chironomidae identified OTUs, (a.) eDNA samples and (b.) community DNA samples, for both amplicons (COIS and COIF). Dashed red lines indicate the rarefaction depth used for analysis (COIS: 4,000 reads). Due to low coverage of COIF eDNA samples (top), this amplicon was excluded from further analysis. x-axis: reads per sample, y-axis: OTU richness (N = 64).

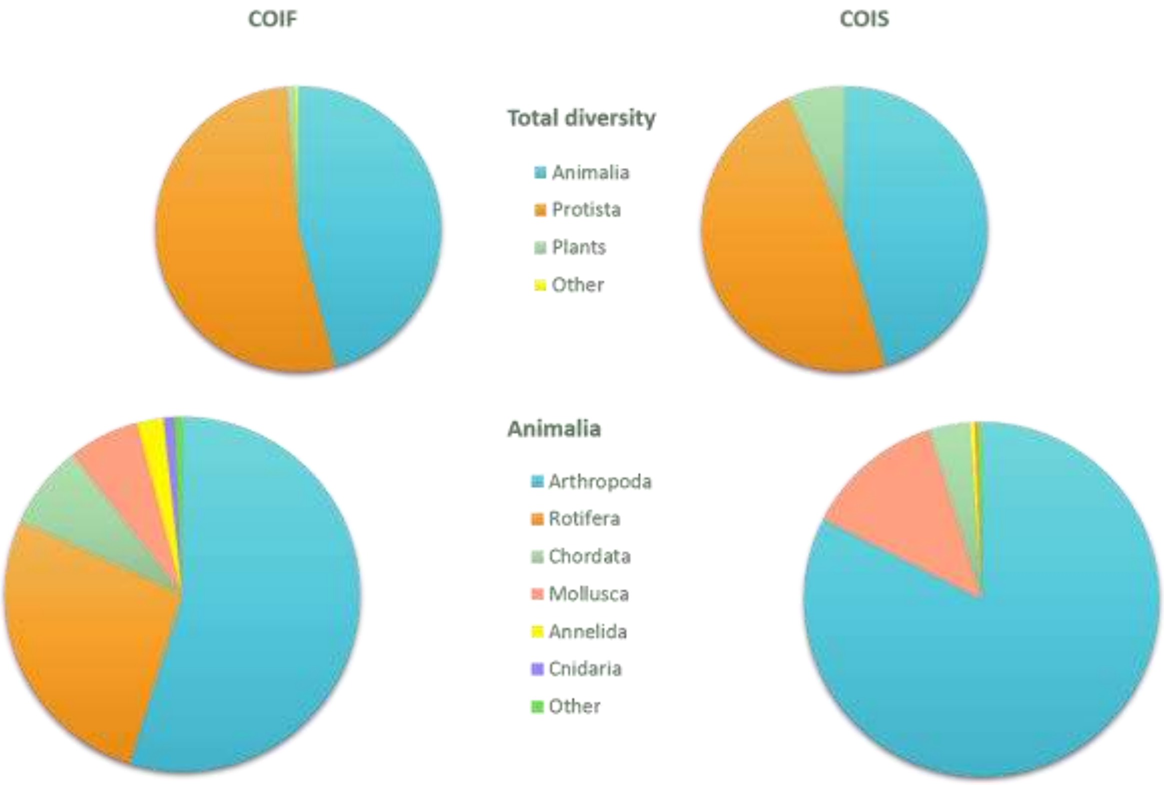


**Supplementary Figure 3 | Summary representation of taxa detected**. Results shown for eDNA samples for both amplicons (COIF, COIS). Top: Kingdoms, bottom: phylum Animalia.

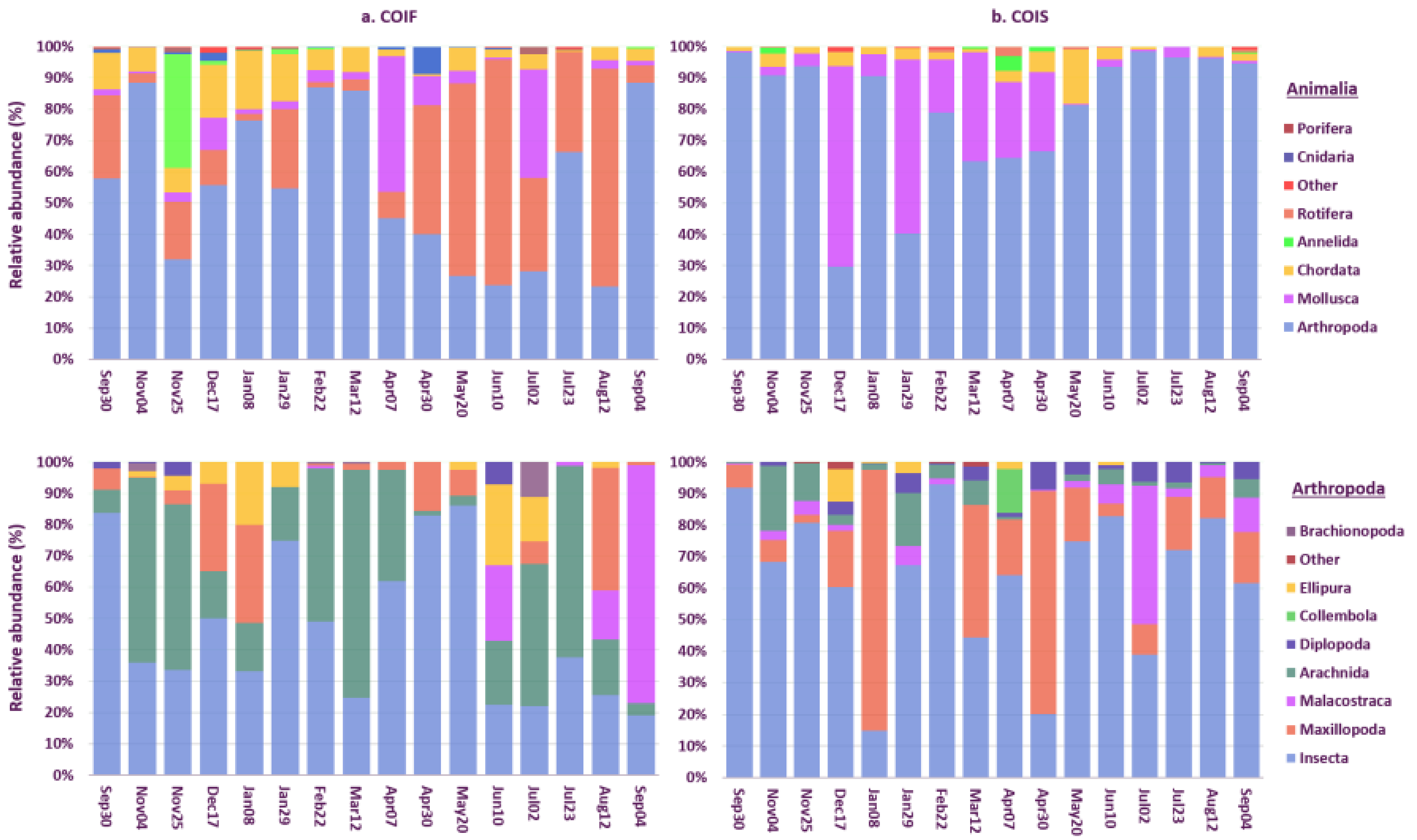


**Supplementary Figure 4 | Histogram presenting taxonomic relative abundance for both amplicons. a**. COIF, **b**. COIS, for all animal (top) and all arthropod (bottom) taxa in eDNA samples through the year (x-axis: sampling dates). All samples were rarefied at 24,914 read depth.

a

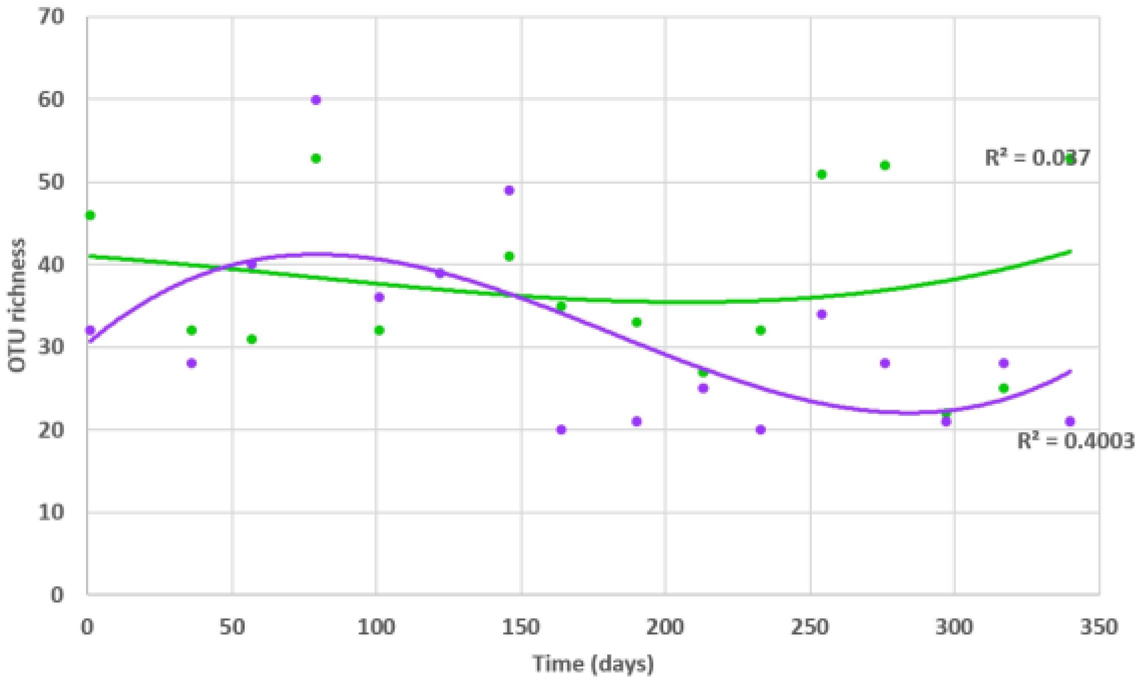


b.

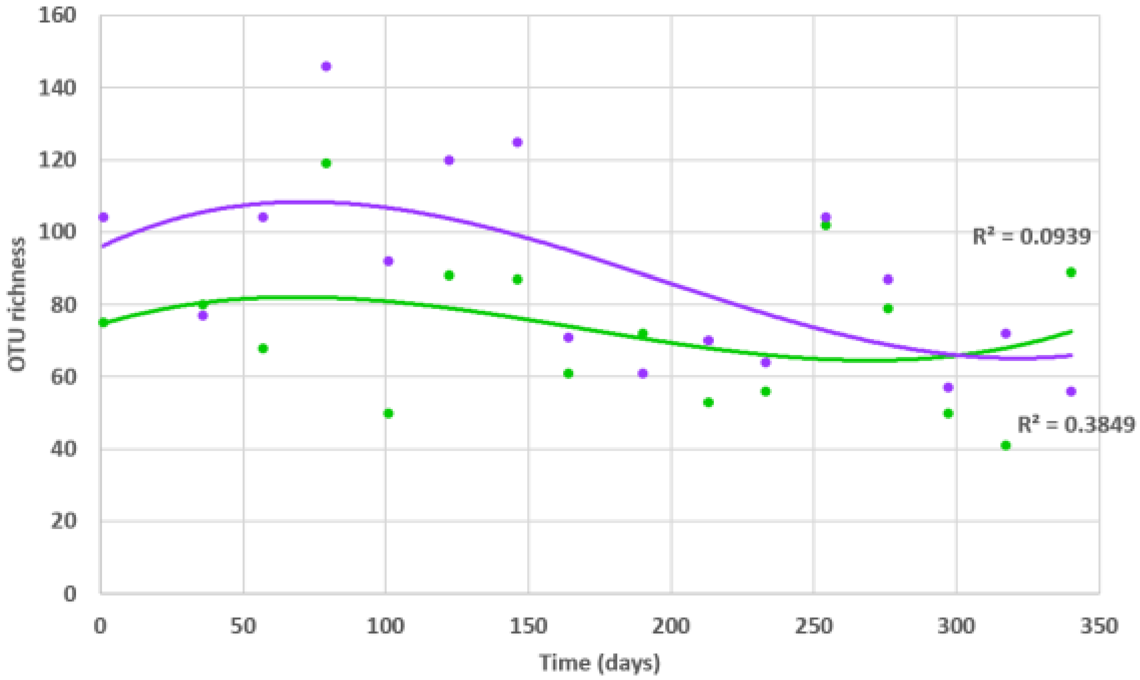


**Supplementary Figure 5 | Yearly trends of OTU richness.**(a) Animal diversity (b) total diversity, detected by eDNA samples for both COIS (green) and COIF (purple). X-axis: time in days (Sep 30th 2014 - Sep 4 2015), y-axis: OTU richness, (a.) COIS: $R^2$=0.037, COIF: $R^2$=0.4003, (b.) COIS: $R^2$=0.0939, COIF: $R^2$=0.3849.

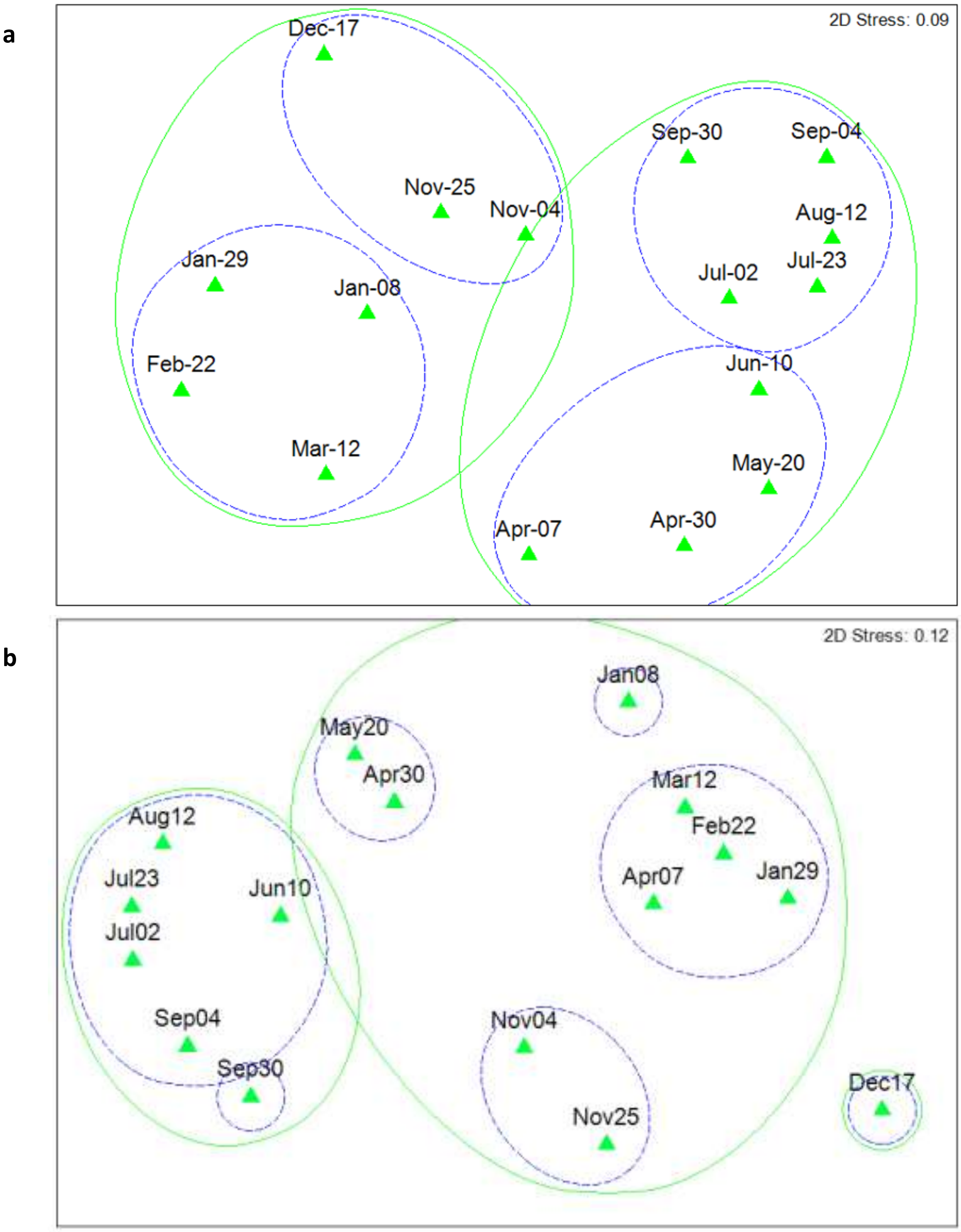


**Supplementary Figure 6 | nMDS plots of β-diversity.** The Sørensen diversity index was calculated for eDNA samples only. **a**: COIF, **b**: COIS (N = 32). Solid green circles: 30% similarity cut-off (corresponding to "winter" –"summer" groups), dashed blue circles: 40% similarity cut-off (N=32).

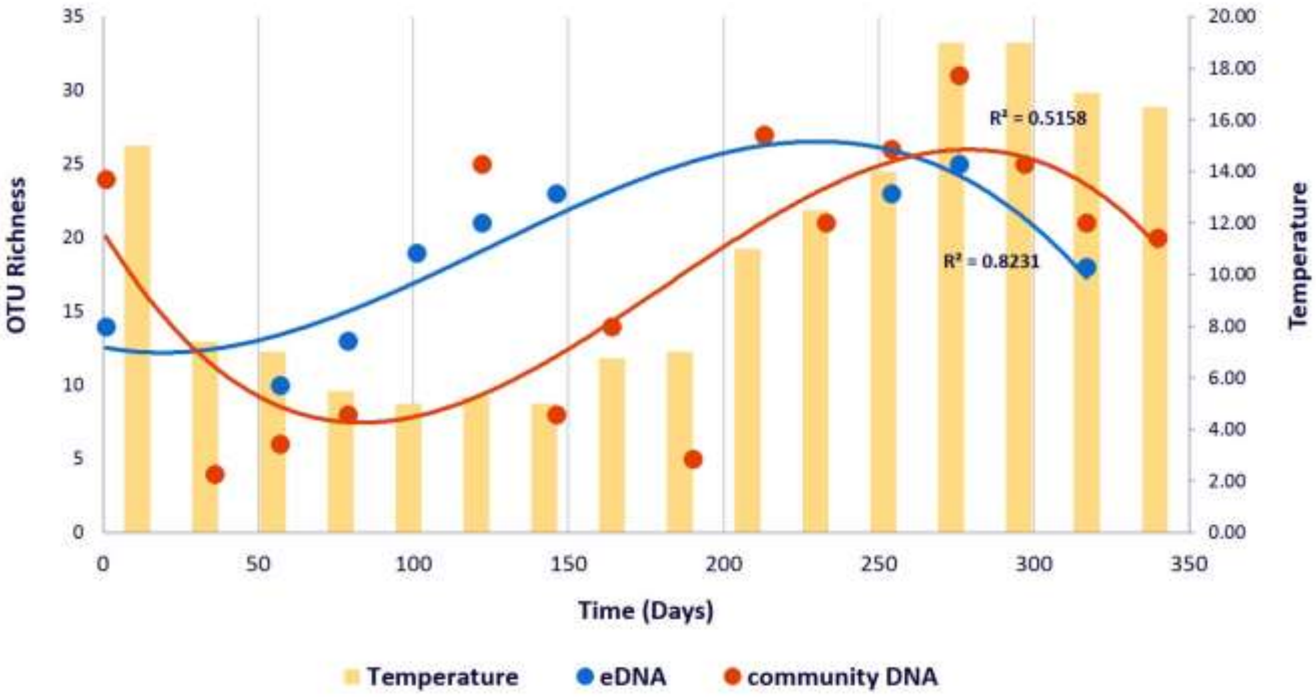


**Supplementary Figure 7 | OTU** richness patterns for Chironomidae OTUs for the COIF amplicon (raw data un-trimmed). Points represent richness values to individual sampling points for eDNA (blue) and community DNA (orange). Best fitted lines from polynomial regressions for eDNA samples (blue) and community DNA (orange), plotted against time (x – axis: Sep. 2013 – Sep 2014), eDNA: $R^2$=0.823, community DNA: $R^2$=0.5158, N=24.

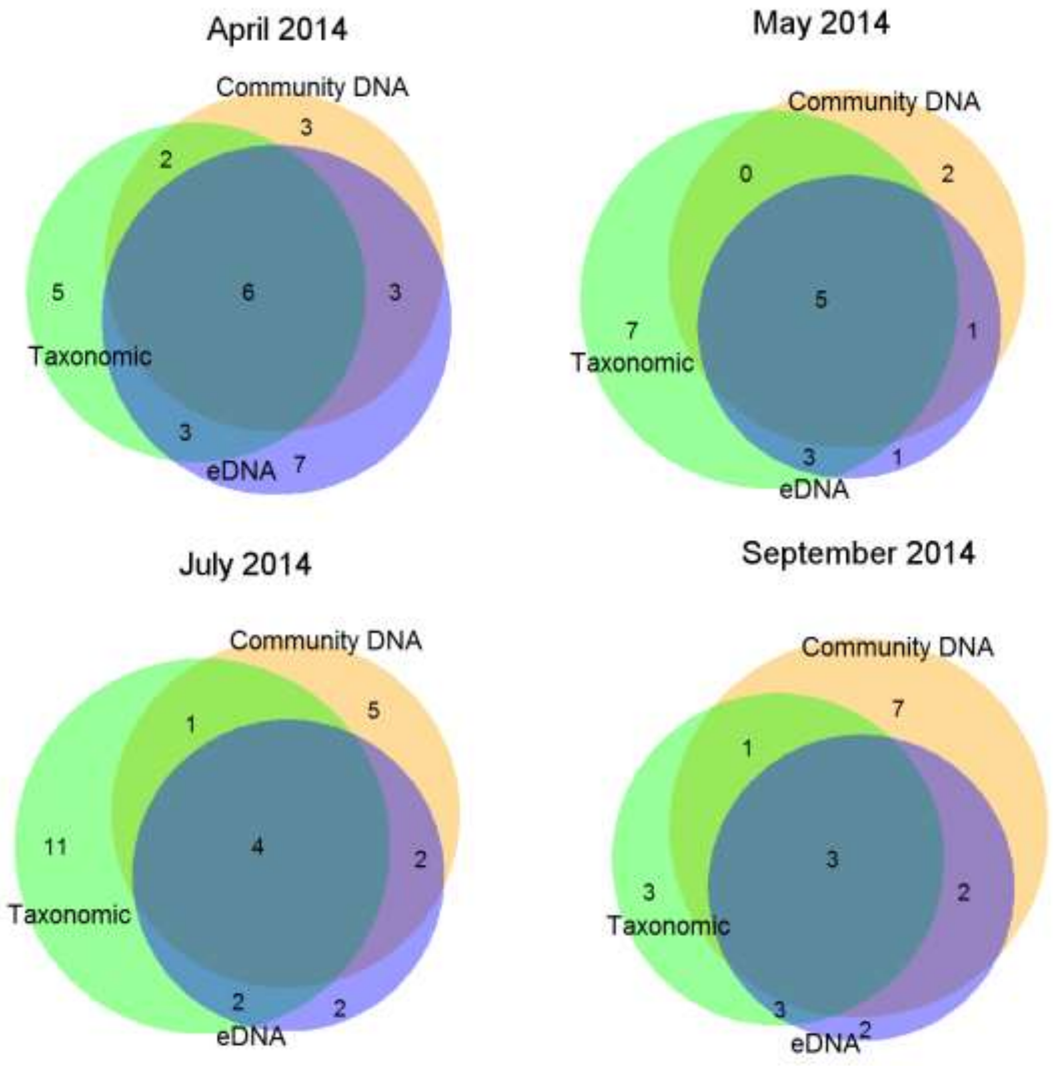


**Supplementary Figure 8 | Venn diagrams of genera richness (individual time points).** Number of Chironomidae genera per sample type (purple: eDNA, orange: community DNA, green: taxonomic data) and the number of genera common between sampling types (overlapped areas). Each plot shows the results for a single time point with the month and year indicated at the top of the respected Venn diagram.

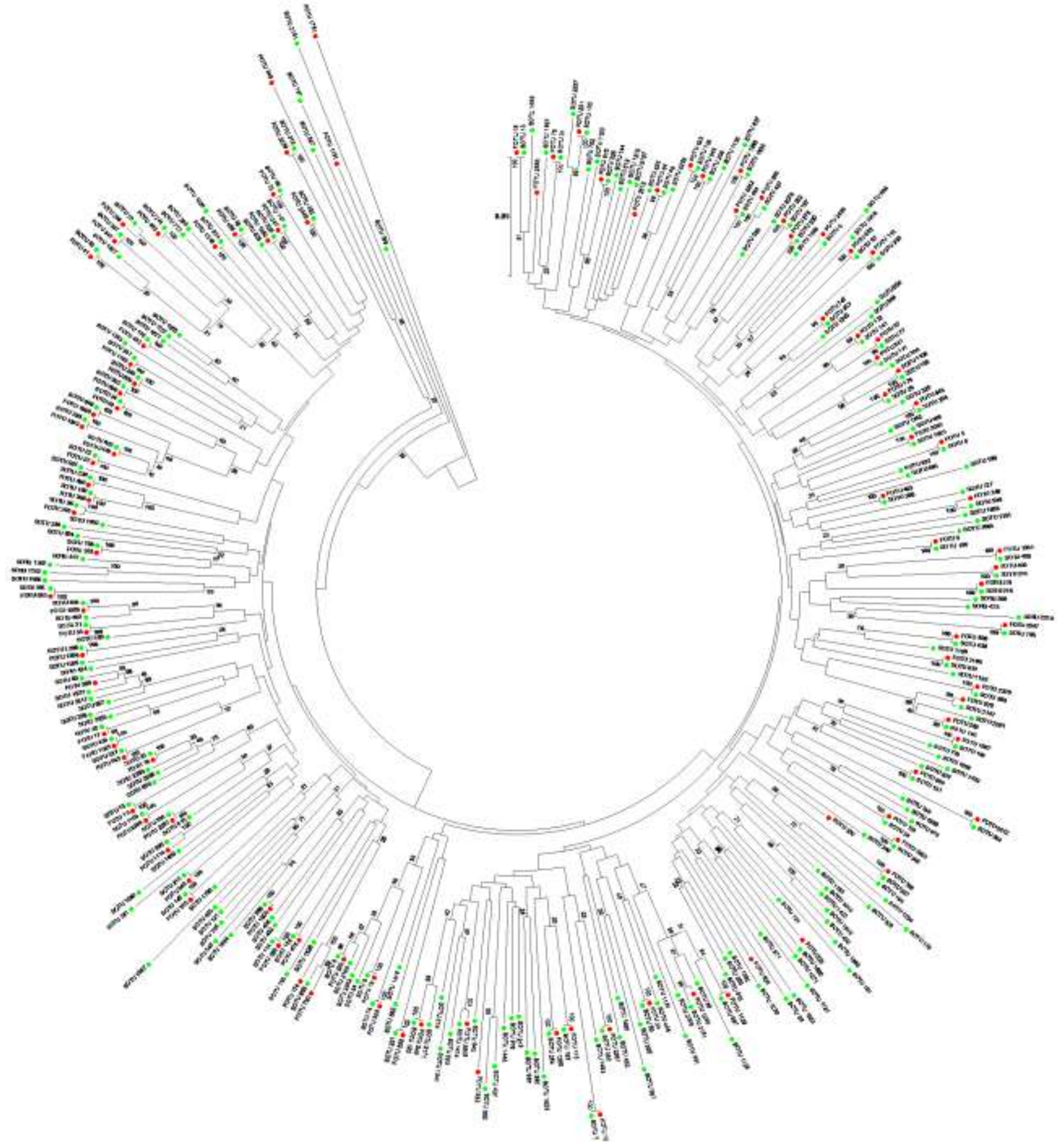

**Supplementary Figure 9 | Neighbor-Joining phylogenetic tree**. The tree comprises all OTUs identified as Chironomidae prior to abundance filtering, for both amplicons (COIF: red markers (FOTU), COIS : green markers (SOTU)). Distances calculated using the p-distance method 1000 bootstrap replications (N = 351).

=

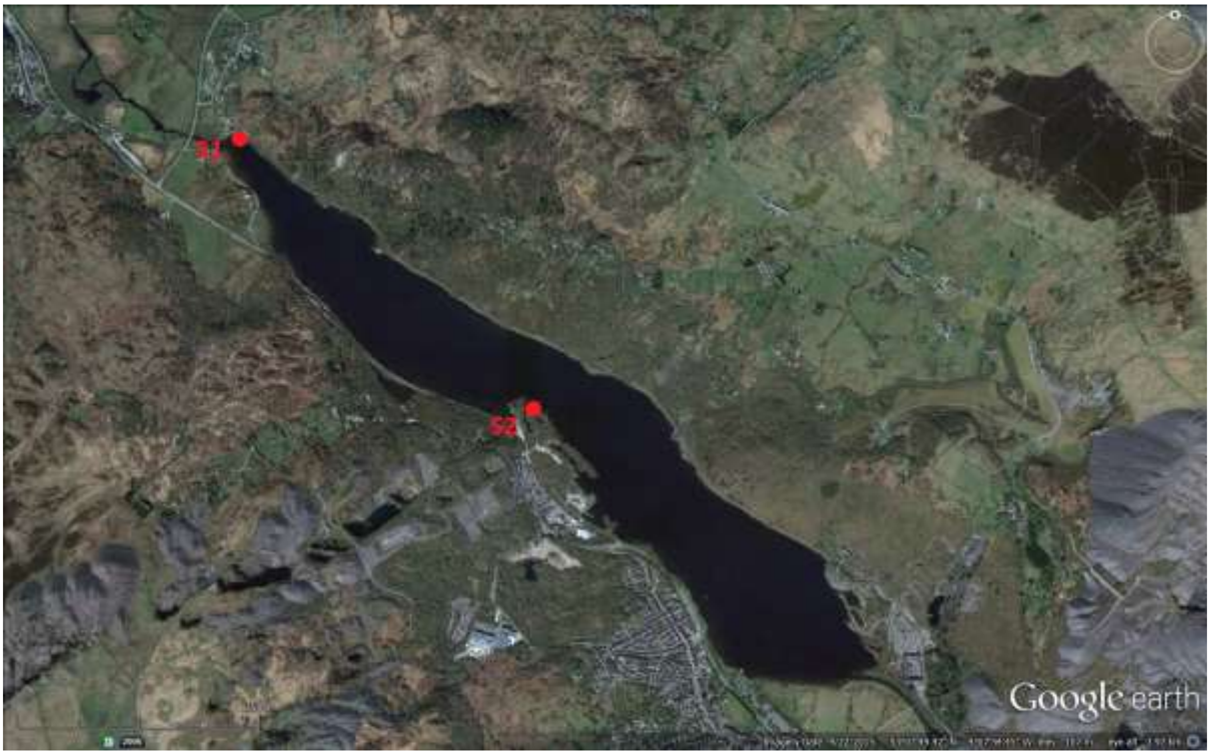


**Supplementary Figure 10 |** Map of Llyn Padarn, N. Wales (UK). Marked with red the two sites used for sample collection (S1: Site 1, NW: 53.139106, -4.153975, S2: Site 2, SW: 53.122414, -4.126761). Google Earth, August 2016. .

## Supplementary Tables

**Supplementary Table 1 | Summary table of number of reads obtained per sample.** Triplicate PCRs from each time point were pooled and sequenced as one. (EXCOI: exuviae community DNA samples, WCOI: water eDNA samples, COIS; 235bp amplicon, COIF: 658bp amplicon).

| #Sample | Sample name | Number of reads | | Sample Type | Collection date | Time point |
|---|---|---|---|---|---|---|
| | | COIS | COIF | | | |
| **1** | 1_EXCOI | 159874 | 383161 | Pupal exuviae | 30/09/2013 | T1 |
| **2** | 2_EXCOI | 464 | 3603 | Pupal exuviae | 04/11/2013 | T2 |
| **3** | 3_EXCOI | 442 | 3808 | Pupal exuviae | 25/11/2013 | T3 |
| **4** | 4_EXCOI | 349 | 2844 | Pupal exuviae | 17/12/2013 | T4 |
| **5** | 5_EXCOI | 203602 | 507 | Pupal exuviae | 08/01/2014 | T5 |
| **6** | 6_EXCOI | 387 | 2406 | Pupal exuviae | 29/01/2014 | T6 |
| **7** | 7_EXCOI | 262 | 365700 | Pupal exuviae | 22/02/2014 | T7 |
| **8** | 8_EXCOI | 411 | 1825 | Pupal exuviae | 12/03/2014 | T8 |
| **9** | 9_EXCOI | 475 | 2755 | Pupal exuviae | 07/04/2014 | T9 |
| **10** | 10_EXCOI | 165644 | 468915 | Pupal exuviae | 30/04/2014 | T10 |
| **11** | 11_EXCOI | 139771 | 363563 | Pupal exuviae | 20/05/2014 | T11 |
| **12** | 12_EXCOI | 289842 | 336948 | Pupal exuviae | 10/06/2014 | T12 |
| **13** | 13_EXCOI | 168006 | 347443 | Pupal exuviae | 02/07/2014 | T13 |
| **14** | 14_EXCOI | 343465 | 15231 | Pupal exuviae | 23/07/2014 | T14 |
| **15** | 15_EXCOI | 489950 | 25608 | Pupal exuviae | 12/08/2014 | T15 |
| **16** | 16_EXCOI | 500181 | 18963 | Pupal exuviae | 04/09/2014 | T16 |
| **17** | 1_WCOI | 240086 | 273799 | Water | 30/09/2013 | T1 |
| **18** | 2_WCOI | 189255 | 260032 | Water | 04/11/2013 | T2 |
| **19** | 3_WCOI | 62109 | 253590 | Water | 25/11/2013 | T3 |
| **20** | 4_WCOI | 288282 | 302474 | Water | 17/12/2013 | T4 |
| **21** | 5_WCOI | 261100 | 346620 | Water | 08/01/2014 | T5 |
| **22** | 6_WCOI | 272002 | 253954 | Water | 29/01/2014 | T6 |
| **23** | 7_WCOI | 157903 | 280711 | Water | 22/02/2014 | T7 |
| **24** | 8_WCOI | 253438 | 263482 | Water | 12/03/2014 | T8 |
| **25** | 9_WCOI | 314163 | 245330 | Water | 07/04/2014 | T9 |
| **26** | 10_WCOI | 282801 | 253024 | Water | 30/04/2014 | T10 |
| **27** | 11_WCOI | 224307 | 154471 | Water | 20/05/2014 | T11 |
| **28** | 12_WCOI | 281971 | 430025 | Water | 10/06/2014 | T12 |
| **29** | 13_WCOI | 252773 | 249347 | Water | 02/07/2014 | T13 |
| **30** | 14_WCOI | 276285 | 285992 | Water | 23/07/2014 | T14 |
| **31** | 15_WCOI | 311891 | 203605 | Water | 12/08/2014 | T15 |
| **32** | 16_WCOI | 309147 | 259862 | Water | 04/09/2014 | T16 |

**Supplementary Table 2 | Positive control contents.** Extracts used for preparation of a positive control sample and taxonomic information of the specimens used for extraction

(species level information was not available for some of the specimens). The last two columns show the success of the amplicons in detecting each extract (**v**: detected, **x**: not detected).

| Positive Control Contents | | | | | Amplicon | |
|---|---|---|---|---|---|---|
| **Number** | **Extract Code** | **Order** | **Family** | **Species** | **COIF** | **COIS** |
| 1 | C_pseudQ24_1 | Amphipoda | Crangonyctidae | *Crangonyx pseudogracilis* | v | v |
| 2 | G_pulexQ29_2 | Amphipoda | Gammaridae | *Gammarus pulex* | v | v |
| 3 | G_marinusQ33_2 | Coleoptera | Gyrinidae | *Gyrinus marinus* | v | v |
| 4 | PA6 | Diptera | Chironomidae | *Chironomidae* sp. | v | v |
| 5 | PA8 | Diptera | Chironomidae | *Chironomidae* sp. | x | v |
| 6 | PA16 | Diptera | Chironomidae | *Chironomidae* sp. | v | v |
| 7 | PA17 | Diptera | Chironomidae | *Chironomidae* sp. | v | v |
| 8 | SERGPSI6_2 | Diptera | Chironomidae | *Sergentia psiloptera* | v | v |
| 9 | ABLAMON2 | Diptera | Chironomidae | *Ablabesmyia monilis* | v | v |
| 10 | CHIRTEN13_1 | Diptera | Chironomidae | *Chironomus tentans* | v | v |
| 11 | CRYPPSI13_1 | Diptera | Chironomidae | *Cryptochironomus psittacinus* | v | v |
| 12 | MONOBAT6A | Diptera | Chironomidae | *Monodiamesa bathyphila* | v | v |
| 13 | CLATATR10A | Diptera | Chironomidae | *Cladotanytarsus atridorsum* | v | v |
| 14 | POLYNUC7B | Diptera | Chironomidae | *Polypedilum nubeculosum* | v | v |
| 15 | E_danicaE130 | Ephemeroptera | Ephemeridae | *Ephemera danica* | x | v |
| 16 | COR1_G18_1 | Gastropoda | Lymnaeidae | *Radix* sp. | v | v |
| 17 | DEV3_G27_1 | Gastropoda | Lymnaeidae | *Radix balthica* | v | v |
| 18 | ANG5_G2_1 | Gastropoda | Planorbidae | *Ancylus fluviatilis* | v | v |
| 19 | A_vortexQ2_2 | Gastropoda | Planorbidae | *Anisus vortex* | v | v |
| 20 | N_glaucaN10 | Hemiptera | Notonectidae | *Notonecta glauca* | v | v |
| 21 | A_aquaticus | Isopoda | Asellidae | *Asellus aquaticus* | x | v |
| 22 | SCO12_T2_1 | Trichoptera | Glossosomatidae | *Agapetus fuscipes* | x | v |
| 23 | COR2_T79_1 | Trichoptera | Goeridae | *Silo pallipes* | v | v |
| 24 | WALE13_T33_1 | Trichoptera | Hydropsychidae | *Hydropsyche instabilis* | v | v |
| 25 | HE1_T4_1 | Trichoptera | Hydroptilidae | *Agraylea sexmaculata* | v | v |
| 26 | YO2_T3_1 | Trichoptera | Hydroptilidae | *Agraylea multipunctata* | v | v |
| 27 | HEA_T37_1 | Trichoptera | Hydroptilidae | *Hydroptila vectis* | v | v |
| 28 | ANG5_T43_1 | Trichoptera | Lepidostomatidae | *Lepidostoma hirtum* | v | v |
| 29 | SCOT2_T27_1 | Trichoptera | Limnephilidae | *Halesus radiatus* | v | v |
| 30 | ANG5_T77_1 | Trichoptera | Leptoceridae | *Athripsodes albifrons* | v | v |

**Supplementary Table 3 | Positive control sequencing results.** Summary table of sequencing results obtained from positive control samples for 235bp COIS and 658bp COIF amplicon.

Shown the number of reads, number of OTUs and relative abundance assigned to our target species (target), unidentified OTUs (unknown) and identified OTUs not present in our target species (Non – target).

| Positive controls | COIS | | | COIF | | |
|---|---|---|---|---|---|---|
| | reads | % | OTUs | reads | % | OTUs |
| **Target** | 547569 | 99.971 | 33 | 393068 | 99.931 | 29 |
| **Unknown** | 18 | 0.003 | 3 | 246 | 0.063 | 16 |
| **Non - Target** | 143 | 0.026 | 14 | 27 | 0.007 | 6 |
| **Total** | 547730 | 100.000 | 50 | 393341 | 100 | 51 |

**Supplementary Table 4 | Summary of eDNA extracts from filter membranes.** Two extractions were performed for each time point which were combined for PCR and sequencing.

| Extract Number | Collection date | Extraction date | Site | DNA concentration (ng/µl) | Time point | DNA concentration - Combined (ng/µl) |
|---|---|---|---|---|---|---|
| **1** | 30/09/2013 | 18/10/2014 | 1 | 72.08 | **T1** | 53 |
| **2** | 30/09/2013 | 18/10/2014 | 2 | 25.34 | | |
| **3** | 04/11/2013 | 18/10/2014 | 1 | 34.92 | **T2** | 37 |
| **4** | 04/11/2013 | 18/10/2014 | 2 | 34.09 | | |
| **5** | 25/11/2013 | 18/10/2014 | 1 | 39.03 | **T3** | 25 |
| **6** | 25/11/2013 | 18/10/2014 | 2 | 11.81 | | |
| **7** | 17/12/2013 | 06/10/2014 | 1 | 24.68 | **T4** | 56 |
| **8** | 17/12/2013 | 06/10/2014 | 2 | 90.58 | | |
| **9** | 08/01/2014 | 06/10/2014 | 1 | 45.5 | **T5** | 46 |
| **10** | 08/01/2014 | 06/10/2014 | 2 | 46.06 | | |
| **11** | 29/01/2014 | 06/10/2014 | 1 | 25.73 | **T6** | 24 |
| **12** | 29/01/2014 | 06/10/2014 | 2 | 21.24 | | |
| **13** | 22/02/2014 | 07/10/2014 | 1 | 58.81 | **T7** | 52 |
| **14** | 22/02/2014 | 07/10/2014 | 2 | 46.87 | | |
| **15** | 12/03/2014 | 06/10/2014 | 1 | 36.62 | **T8** | 36 |
| **16** | 12/03/2014 | 06/10/2014 | 2 | 37.69 | | |
| **17** | 07/04/2014 | 07/10/2014 | 1 | 77.77 | **T9** | 76 |
| **18** | 07/04/2014 | 07/10/2014 | 2 | 75.19 | | |
| **19** | 30/04/2014 | 06/10/2014 | 1 | 47.33 | **T10** | 49 |
| **20** | 30/04/2014 | 06/10/2014 | 2 | 49.72 | | |
| **21** | 20/05/2014 | 18/10/2014 | 1 | 80.05 | **T11** | 68 |
| **22** | 20/05/2014 | 18/10/2014 | 2 | 52.55 | | |
| **23** | 10/06/2014 | 07/10/2014 | 1 | 44.49 | **T12** | 47 |
| **24** | 10/06/2014 | 07/10/2014 | 2 | 50.33 | | |
| **25** | 02/07/2014 | 18/10/2014 | 1 | 37.74 | **T13** | 48 |
| **26** | 02/07/2014 | 18/10/2014 | 2 | 44.94 | | |
| **27** | 23/07/2014 | 18/10/2014 | 1 | 66.18 | **T14** | 62 |
| **28** | 23/07/2014 | 18/10/2014 | 2 | 45.93 | | |
| **29** | 12/08/2014 | 18/10/2014 | 1 | 90.28 | **T15** | 68 |
| **30** | 12/08/2014 | 18/10/2014 | 2 | 35.4 | | |
| **31** | 04/09/2014 | 18/10/2014 | 1 | 80.02 | **T16** | 65 |
| **32** | 04/09/2014 | 18/10/2014 | 2 | 41.33 | | |

**Supplementary Table 5 | Summary of DNA extracts from exuviae community samples.**

| Extract Number | Collection date | Extraction date | DNA concentration (ng/µl) | Time point | Method |
|---|---|---|---|---|---|
| 1 | 30/09/2013 | 23/11/2014 | 36.98 | E1 | QIAmp Blood Maxi |
| 2 | 04/11/2013 | 19/11/2014 | 9.81 | E2 | Qiagen B & T Kit |
| 3 | 25/11/2013 | 19/11/2014 | 6.59 | E3 | Qiagen B & T Kit |
| 4 | 17/12/2013 | 19/11/2014 | 12.26 | E4 | Qiagen B & T Kit |
| 5 | 08/01/2014 | 19/11/2014 | 9.57 | E5 | Qiagen B & T Kit |
| 6 | 29/01/2014 | 19/11/2014 | 9.01 | E6 | Qiagen B & T Kit |
| 7 | 22/02/2014 | 19/11/2014 | 6.13 | E7 | Qiagen B & T Kit |
| 8 | 12/03/2014 | 19/11/2014 | 12.15 | E8 | Qiagen B & T Kit |
| 9 | 07/04/2014 | 19/11/2014 | 16.5 | E9 | Qiagen B & T Kit |
| 10 | 30/04/2014 | 23/11/2014 | 35.7 | E10 | QIAmp Blood Maxi |
| 11 | 20/05/2014 | 23/11/2014 | 31.31 | E11 | QIAmp Blood Maxi |
| 12 | 10/06/2014 | 23/11/2014 | 30.15 | E12 | QIAmp Blood Maxi |
| 13 | 02/07/2014 | 23/11/2014 | 18.15 | E13 | QIAmp Blood Maxi |
| 14 | 23/07/2014 | 23/11/2014 | 19.9 | E14 | QIAmp Blood Maxi |
| 15 | 12/08/2014 | 23/11/2014 | 19.89 | E15 | QIAmp Blood Maxi |
| 16 | 04/09/2014 | 23/11/2014 | 25.42 | E16 | QIAmp Blood Maxi |

**Supplementary Table 6 |** Primers used for library preparation. Round 1: forward / reverse universal tail and template specific primer. A multi N region inserted in forward primer to assist cluster formation. Round 2: a forward or reverse Illumina adapter and an i5 or i7 Nextera index with the appropriate universal tail.

| Primer pair | Round 1 | Direction |
|---|---|---|
| **LCO1490** | **Forward Universal tail** **Template specific primer**<br>ACACTCTTTCCCTACACGACGCTCTTCCGATCT **NNNNN** GGTCAACAAATCATAAAGATATTGG | **Forward** |
| **HC02198** | **Reverse Universal tail** **Template specific primer**<br>GTGACTGGAGTTCAGACGTGTGCTCTTCCGATCTTAAACTTCAGGGTGACCAAAAAATCA | **Reverse** |
| **COI_A_rev** | **Reverse Universal tail** **Template specific primer**<br>GTGACTGGAGTTCAGACGTGTGCTCTTCCGATCTCARAAWCTTATATTATTTATTCGDGG | **Reverse** |
| | **Round 2** | |
| **All Forward** | **P5 Illumina adapter** **Index 2 (i5)** **Forward Universal tail**<br>5' AATGATACGGCGACCACCGAGATCTACAC - i5 Index - ACACTCTTTCCCTACACGACGCTC 3' | **Forward** |
| **All Reverse** | **P7 Illumina adapter** **Index 1 (i7)** **Reverse Universal tail**<br>5' CAAGCAGAAGACGGCATACGAGAT - i7 Index - GTGACTGGAGTTCAGACGTGTGCTC 3' | **Reverse** |

## Supplementary References


1. Wilson, R. & Ruse, L. *A guide to the identification of genera of chironomid pupal exuviae occurring in Britain and Ireland*. (Freshwater Biological Association Publication 13, Ambleside, UK., 2005).
2. Ruse, L. Lake acidification assessed using chironomid pupal exuviae. *Fundam. Appl. Limnol. / Arch. für Hydrobiol.* **178,** 267–286 (2011).
3. Armitage, P. D., Pinder, L. C. & Cranston, P. The Chironomidae: biology and ecology of non-biting midges. (Chapman and Hall, 1995).
4. Renshaw, M. A., Olds, B. P., Jerde, C. L., Mcveigh, M. M. & Lodge, D. M. The room temperature preservation of filtered environmental DNA samples and assimilation into a phenol-chloroform-isoamyl alcohol DNA extraction. *Mol. Ecol. Resour.* **15,** 168–176 (2015).
